# Ipsilateral Repeated Bout Effect Across Heterologous Muscle Groups: Eccentric Knee Extensor Conditioning Enhances Elbow Flexor Recovery in Young Women

**DOI:** 10.3390/life15060919

**Published:** 2025-06-06

**Authors:** Fu-Shun Hsu, Chung-Chan Hsieh, Chia-Yu Tang, Chang-Chi Lai, Yu-Jui Li, Yun-Chung Tseng, Szu-Kai Fu

**Affiliations:** 1 Department of Exercise and Health Sciences, College of Kinesiology, University of Taipei, Taipei 111036, Taiwan; dba71@tpech.gov.tw (F.-S.H.); t51682000@yahoo.com.tw (C.-C.H.); sportsinjury0406@gmail.com (C.-C.L.); 2 Department of Urology, Yangming Branch, Taipei City Hospital, Taipei 111024, Taiwan; 3 Department of Urology, College of Medicine, National Taiwan University, Taipei 100229, Taiwan; 4 Graduate Institute of Sports Training, College of Kinesiology, University of Taipei, Taipei 111036, Taiwan; jimmy_lin19@yahoo.com.tw; 5 Department of Recreation and Sports Management, College of Kinesiology, University of Taipei, Taipei 111036, Taiwan; li542058@utaipei.edu.tw; 6 Department of Mathematical Sciences, College of Science, National Chengchi University, Taipei 11605, Taiwan; leo08102004@gmail.com

**Keywords:** repeated bout effect, eccentric exercise, cross-education, proprioception, preconditioning, neuroinflammation, ipsilateral transfer, flow effect

## Abstract

This study investigated whether prior eccentric exercise of knee extensors could attenuate muscle damage in ipsilateral elbow flexors, supporting the presence of an ipsilateral repeated bout effect (IL-RBE) across heterogeneous muscle groups. Sixteen young women were randomized into an intervention group (NL/NU) and a control group (C/NU). The NL/NU group performed eccentric knee extensor exercise 14 days before elbow flexor eccentric loading. Compared to the C/NU group, the NL/NU group exhibited an earlier return to baseline in muscle stiffness (D3: NL/NU = 1.14 ± 0.05 vs. Pre = 0.96 ± 0.03 m/s), joint release angle at 30° (D3: NL/NU = 22.79 ± 1.02 vs. Pre = 24.46 ± 0.87°), and joint release angle at 45° (D2: NL/NU = 37.75 ± 1.38 vs. Pre = 38.83 ± 0.87°), indicating a faster recovery trend in these specific neuromuscular and morphological measures. These results suggest that prior remote eccentric loading induces systemic and neuromuscular adaptations, facilitating improved functional recovery. The findings expand IL-RBE applicability to heterologous muscles within the same limb and support its integration into training and rehabilitation protocols.

## 1. Introduction

Resistance exercise training is widely applied in various populations for enhancing athletic performance and rehabilitation outcomes. It primarily encompasses concentric, eccentric, and isometric muscle contractions. Particularly, individuals unaccustomed to regular physical activity may experience subtle muscle fiber damage following unaccustomed resistance exercises, often manifesting as delayed onset muscle soreness (DOMS). DOMS typically peaks 24–72 h post-exercise and may require 7–10 days or longer for complete recovery [1]. The primary mechanisms underlying exercise-induced muscle damage (EIMD) include mechanical stress, metabolic stress, or a combination of both [2]. Previous studies reported that eccentric contractions, in comparison to concentric or isometric contractions, result in more pronounced muscle damage, characterized by reductions in maximal voluntary contraction strength (MVC), decreased range of motion (ROM), increased creatine kinase (CK) activity, limb swelling, and impaired proprioception [3,4,5]. The repeated bout effect (RBE) describes the phenomenon where a second bout of eccentric exercise, performed days to weeks after an initial bout, leads to significantly reduced muscle damage compared to the initial exposure. When this protective effect is observed in the same muscle group, it is termed the ipsilateral repeated bout effect (IL-RBE) [6]. This adaptation is characterized by less severe reductions in MVC and ROM, lower DOMS, smaller increases in CK activity, reduced limb circumference swelling (CIR), and faster recovery [7,8]. IL-RBE appears maximal when the second bout occurs 2–4 weeks after the initial exercise and may persist for up to 6 months, albeit diminishing over longer intervals [9,10]. Interestingly, performing an eccentric exercise bout on one limb may confer protective effects on the contralateral limb, termed the contralateral repeated bout effect (CL-RBE). For instance, previous research indicated that an initial bout of eccentric elbow flexor exercises reduced muscle damage symptoms in the contralateral elbow flexors when performed weeks later [8,10]. However, the duration between exercise bouts significantly influences the presence and magnitude of CL-RBE [8]. The existence of CL-RBE has primarily been established within homologous muscle groups across limbs, but its effectiveness between heterogeneous muscle groups (e.g., upper limb to lower limb) remains inconclusive. Some studies have demonstrated no significant protective effect between different muscle groups across limbs [11]. Conversely, other studies have shown possible systemic effects, where muscle damage induced by activities such as downhill running affected performance in distant, unrelated muscle groups [12]. Such discrepancies highlight the complexity of the mechanisms underlying CL-RBE. Given that knee extensors and elbow flexors are among the most functionally relevant muscle groups in daily activities (e.g., walking, running, lifting), understanding the interaction of cross-education effects between these muscle groups is crucial. Additionally, the majority of previous studies focused primarily on males or specific age groups, raising questions regarding generalizability to other populations, including females [13]. Moreover, fluctuations in hormonal levels during the menstrual cycle can influence neuromuscular performance [14]. Therefore, our study design included only eumenorrheic women not using hormonal contraception, with testing scheduled outside the early follicular phase to minimize hormonal variation.

Therefore, further investigations are necessary to elucidate the mechanisms, practical implications, and scope of CL-RBE across different muscle groups and populations. Given that most previous RBE and cross-education studies were conducted in male or mixed-sex populations, the current study specifically recruited young adult females to address the underrepresentation of this group in the literature. Hormonal influences such as estradiol may modulate muscle stiffness and inflammatory responses, and while its direct protective effect on EIMD is modest in eumenorrheic women [13], sex-related neuromuscular differences may still influence the presentation and recovery profile of RBE-related adaptations. Thus, a better understanding of IL-RBE in females is essential to broaden the applicability of eccentric preconditioning across sexes.

The present study aimed to examine whether an initial bout of eccentric exercise targeting the knee extensors provides protective effects on subsequent muscle damage in the ipsilateral elbow flexors. By investigating IL-RBE across heterologous muscle groups in young women, this study sought to provide foundational evidence for sex-specific training and rehabilitation strategies, and to clarify the systemic and localized mechanisms underlying eccentric-induced neuromuscular adaptations.

## 2. Materials and Methods

### 2.1. Study Design

**Figure 1:**
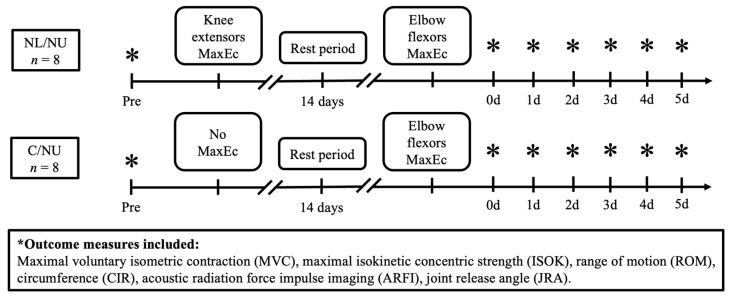
Study design flow chart. Note: NL/NU = Non-dominant lower limb eccentric exercise followed by non-dominant upper limb eccentric exercise; C/NU = Control followed by non-dominant upper limb eccentric exercise; MaxEc = Maximal eccentric exercise.

This study employed a randomized, controlled design to examine the ipsilateral repeated bout effect (IL-RBE) across heterogeneous muscle groups in young women. Participants were randomly assigned to two groups (*n* = 8 per group): the NL/NU group, who performed eccentric knee extensor exercise followed by eccentric elbow flexor exercise 14 days later; and the C/NU group, who received no lower-limb intervention and completed only the elbow flexor eccentric exercise. Outcome measurements were collected at seven time points: baseline (Pre), immediately after the elbow flexor exercise (0 day), and on days 1 through 5 post-exercise (D1–D5). A schematic illustration of the study design and testing timeline is presented in Figure 1.

A priori power analysis was performed using G*Power 3.1.9.7 to estimate the required sample size. Based on a two-way, mixed-design ANOVA with repeated measures (within–between interaction), and assuming a moderate effect size (f = 0.40), alpha level α = 0.05, and statistical power (1-β) = 0.80, the estimated sample size required was approximately 102 participants (51 per group). However, given the exploratory nature of this study and practical constraints, we adopted a feasibility-based sample size of 16 participants (8 per group), which is consistent with previous pilot studies investigating repeated bout effects in similar exercise settings. This sample size allows for the initial evaluation of potential ipsilateral cross-adaptation trends and provides foundational data for planning future studies with larger statistical power.

### 2.2. Participants

Sixteen healthy adult women aged 20 to 30 years (mean ± SD: 23.06 ± 2.43 years; height: 162.10 ± 4.40 cm; body mass: 56.93 ± 4.50 kg; body fat: 25.50 ± 4.13%) participated in this study. Inclusion criteria included absence of cardiovascular diseases, pacemakers, musculoskeletal injuries in the upper or lower limbs, and no regular engagement in exercise training or heavy labor during the preceding six months. To control for potential hormonal influences, only eumenorrheic participants who were not taking any form of hormonal contraception were included. Data collection was scheduled to avoid the early follicular phase (days 1–5 of the menstrual cycle), during which estrogen and progesterone levels are lowest and neuromuscular performance may be reduced. All participants were informed of the study’s purpose, procedures, potential risks, and benefits, and provided written informed consent prior to participation. This study was conducted in accordance with the Declaration of Helsinki and was approved by the Research Ethics Committee of National Taiwan Normal University (Protocol No. 202305HM061; approval date: 2 July 2023). During the experimental period, participants were instructed to refrain from additional exercise, vitamin or high-protein supplementation, and any form of physical therapy.

### 2.3. Experimental Procedures

Participants were randomly assigned to two groups: NL/NU and C/NU. The NL/NU group performed two maximal voluntary eccentric contraction (MaxEc) sessions with a 14-day interval, starting with 60 repetitions of knee extensor eccentric exercise (MaxEc1) followed by 30 repetitions of elbow flexor eccentric exercise (MaxEc2). The C/NU (control) group performed only the elbow flexor eccentric exercise session (30 repetitions). Knee extensor exercises consisted of 10 sets of 6 repetitions, while elbow flexor exercises comprised 5 sets of 6 repetitions. Each contraction lasted 3 s at an angular velocity of 30°/s, with a 15 s rest between repetitions and a 2 min rest between sets. Outcome measures were assessed at baseline (pre-exercise), immediately after exercise (0 days), and at 1, 2, 3, 4, and 5 days post-exercise.

### 2.4. Familiarization

Participants underwent a familiarization session involving (1) overview of assessment procedures, (2) practice of maximal eccentric contractions, and (3) rehearsal of measurement protocols.

### 2.5. Maximal Eccentric Exercise

A Biodex System 4 Pro dynamometer (Biodex Medical Systems, Shirley, NY, USA) facilitated eccentric exercises. For elbow flexors, participants were seated with the shoulder positioned at 45° flexion and 0° abduction, and the elbow joint starting at 90° of flexion (0° representing full elbow extension). For knee extensors, exercises were performed in a prone position with the knee joint starting at 90° of flexion (0° being full knee extension). Participants maximally resisted the dynamometer arm for 3 s at an angular velocity of 30°/s during each repetition, moving from 90° flexion to full extension (0°) in both elbow flexion and knee extension exercises.

### 2.6. Outcome Measures

Outcome assessments were categorized into three domains: (1) strength measurements, (2) joint and muscle morphology assessments, and (3) neuromuscular control. Specifically, the measured variables included MVC, maximal isokinetic concentric strength (ISOK), ROM, CIR, muscle stiffness, and joint release angle (JRA). All assessments were performed by the same trained examiner, who was blinded to group allocation throughout the study to reduce measurement bias.

#### 2.6.1. Strength Measurements

##### Maximal Voluntary Isometric Contraction

MVC was assessed using a Biodex System 4 Pro dynamometer (Biodex Medical Systems, Shirley, NY, USA). Participants performed three maximal isometric contractions, each lasting 3 s with a 45 s rest between trials. The highest recorded torque was used for analysis.

##### Maximal Isokinetic Concentric Strength

ISOK was assessed using the same Biodex System 4 Pro dynamometer (Biodex Medical Systems, Shirley, NY, USA), operated with Biodex Advantage Software, version 4.56. Participants adopted the same seated posture as in the MVC assessment, with the trunk, pelvis, and tested limb securely strapped to the chair. The shoulder was positioned at 45° flexion and 0° abduction. The elbow joint started at 0° of flexion (full extension) and moved towards 90° flexion during the contraction. Participants performed five maximal concentric contractions each at two angular velocities (60°/s and 180°/s), with a 2 min rest interval between velocity conditions. The highest peak torque recorded at each velocity was used for analysis.

#### 2.6.2. Joint and Muscle Morphology Assessments

##### Range of Motion

ROM was evaluated using a standard plastic goniometer (Lafayette Instrument Co., Lafayette, IN, USA), calculated as the difference between maximal joint flexion and extension angles.

##### Limb Circumference

CIR was measured at the mid-belly of the targeted muscle group using a flexible tape measure (commonly used in field assessment; no specific manufacturer). Three readings were taken per day and averaged for analysis.

##### Muscle Stiffness

Muscle stiffness was assessed using acoustic radiation force impulse imaging (ARFI) via an ACUSON S2000 ultrasound system (Siemens Healthcare, Erlangen, Germany) equipped with a 9L4 linear-array transducer (4–9 MHz). Shear wave velocity (m/s), a reliable indicator of tissue elasticity, was measured at the mid-belly of the target muscle. For each time point, three transverse plane images were acquired and averaged to obtain the final value.

#### 2.6.3. Neuromuscular Control

##### Joint Release Angle

Participants were blindfolded to eliminate visual feedback and instructed to stop limb movement as quickly as possible after the release of the dynamometer arm. Measurements were taken at target angles of 30°, 45°, and 60°.

### 2.7. Data Availability

All datasets generated and analyzed during the current study are available from the corresponding author upon reasonable request.

### 2.8. Statistical Analysis

Data analyses were conducted using SPSS version 20.0 (IBM Corp., Armonk, NY, USA). Descriptive statistics are presented as mean ± standard deviation (SD). A two-way, mixed-design ANOVA (group × time) was performed to examine the interaction and main effects across time points between the NL/NU and C/NU groups. When no significant interaction was observed, within-group recovery patterns were further analyzed using one-way, repeated measures ANOVA followed by LSD post-hoc tests, comparing each post-exercise time point (D0–D5) against the baseline (Pre) within each group. The time point at which outcome measures returned to values not significantly different from baseline was used to determine the relative recovery timeline of each group. The level of statistical significance was set at α = 0.05.

## 3. Results

At baseline (Pre), there were no statistically significant differences between the NL/NU and C/NU groups in any of the outcome variables, indicating homogeneity across groups prior to the intervention (*p* > 0.05 for all comparisons). This confirms that both groups were comparable in elbow flexor strength, ROM, CIR, muscle stiffness, and neuromuscular control (Table 1).

Following the elbow flexor eccentric exercise, both groups demonstrated acute declines in MVIC and concentric strength (60°/s and 180°/s), accompanied by reduced ROM, increased CIR and stiffness, and impairments in JRA. However, the NL/NU group—having undergone a prior bout of knee extensor eccentric exercise—exhibited a more favorable recovery profile. This was particularly evident in the faster restoration of peak torque and muscle stiffness values, as well as earlier normalization of JRA measurements compared to the C/NU group. Although no significant group × time interaction was detected, within-group comparisons revealed that the NL/NU group returned to baseline levels earlier across multiple outcome domains. Specifically, muscle stiffness and ROM showed normalization by Day 3 or Day 4 in the NL/NU group, whereas similar recovery in the C/NU group was observed later (Day 4 or Day 5). These findings suggest an earlier recovery trend in the NL/NU group based on within-group statistical comparisons.

These findings suggest that a prior eccentric exercise stimulus in a different (but ipsilateral) muscle group may confer a protective cross-adaptation benefit to subsequent upper limb muscle performance (Table 1).

### 3.1. Recovery of Strength Outcomes

**Figure 2:**
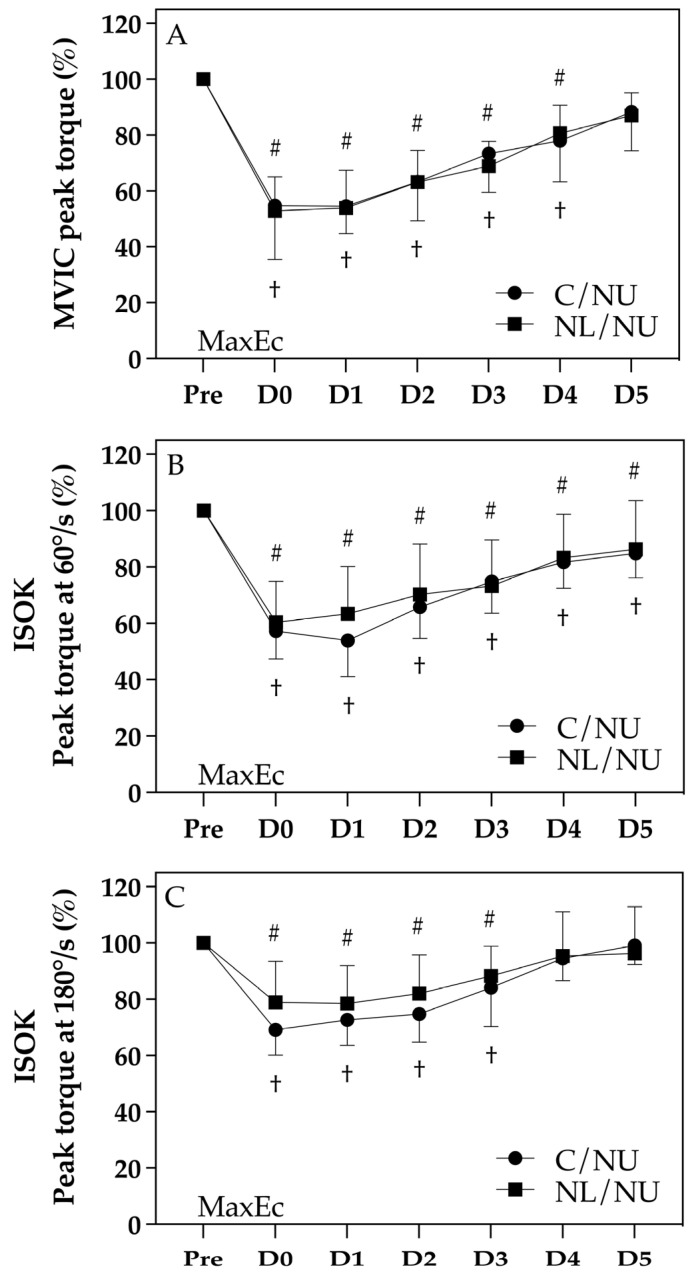
Strength measurement. Note: † indicates significant difference from Pre in the C/NU group (*p* < 0.05); # indicates significant difference from Pre in the NL/NU group (*p* < 0.05). C/NU = control followed by non-dominant upper limb eccentric exercise; NL/NU = non-dominant lower limb eccentric exercise followed by non-dominant upper limb eccentric exercise. MaxEc = maximal eccentric exercise. (**A**) Changes in maximal voluntary isometric contraction (MVIC) peak torque (%); (**B**) Changes in maximal isokinetic concentric strength at 60°/s (%); (**C**) Changes in maximal isokinetic concentric strength at 180°/s (%).

Figure 2 displays the normalized changes in elbow flexor strength across both groups at baseline (Pre), immediately after exercise (D0), and up to 5 days post-exercise (D1–D5), including (A) MVIC, (B) concentric torque at 60°/s, and (C) concentric torque at 180°/s. While no significant group × time interaction was observed (*p* > 0.05), distinct within-group patterns emerged based on the symbol annotations.

In all three strength indicators, both groups showed significant declines from baseline following MaxEc († or #, *p* < 0.05). However, recovery trajectories differed between groups. In the C/NU group, strength remained significantly lower than baseline until D4 or D5 in most measures (†), whereas the NL/NU group returned to baseline levels earlier in the recovery period (by D3 or D4), particularly for MVIC and torque at 60°/s (#). This suggests that the NL/NU group exhibited superior recovery capacity, potentially due to a protective effect conferred by the prior eccentric exercise of the knee extensors.

These findings support the presence of an IL-RBE, indicating that eccentric loading of the knee extensors may facilitate faster neuromuscular recovery in the ipsilateral elbow flexors.

### 3.2. Changes in Joint and Muscle Morphology

**Figure 3:**
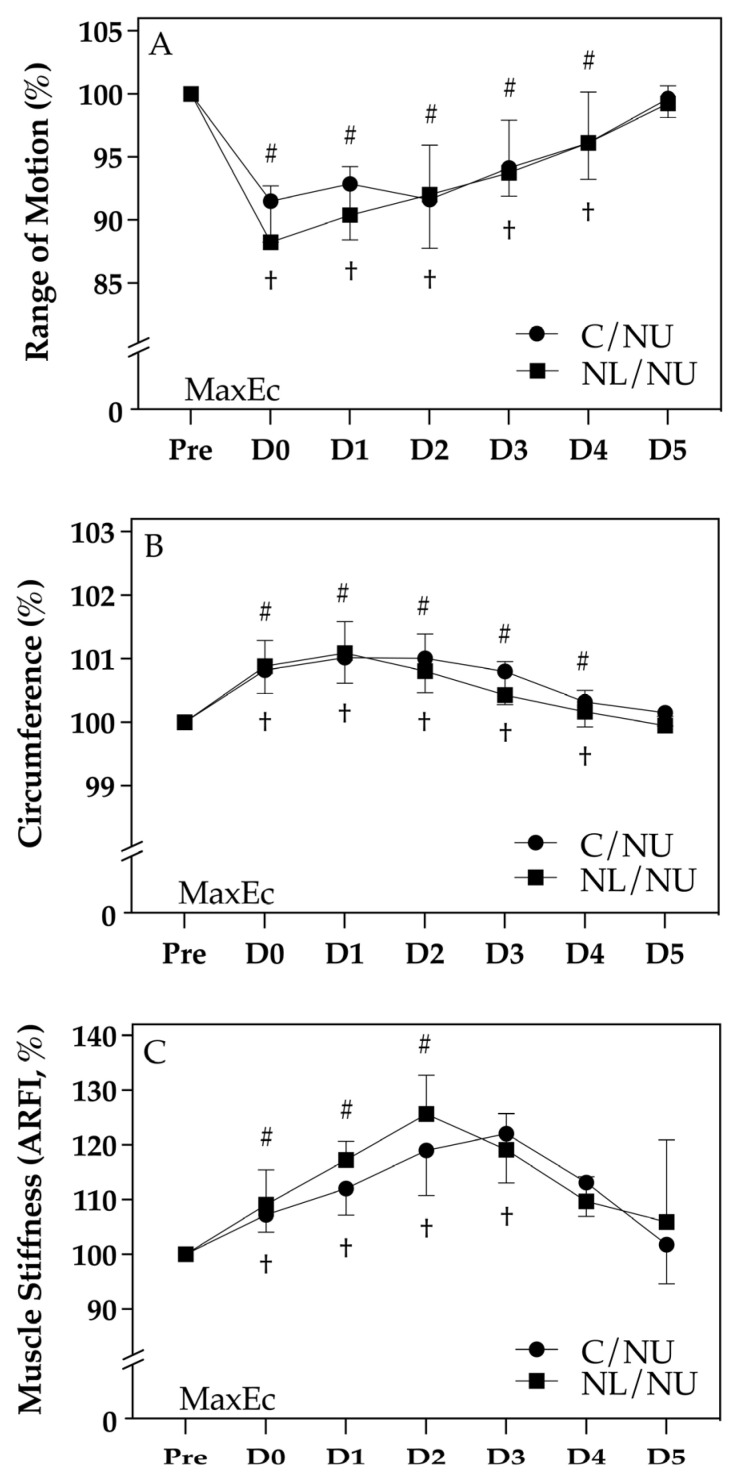
Joint and muscle morphology assessments. Note: † indicates significant difference from Pre in the C/NU group (*p* < 0.05); # indicates significant difference from Pre in the NL/NU group (*p* < 0.05). C/NU = control followed by non-dominant upper limb eccentric exercise; NL/NU = non-dominant lower limb eccentric exercise followed by non-dominant upper limb eccentric exercise. MaxEc = maximal eccentric exercise. ARFI = acoustic radiation force impulse imaging. (**A**) Changes in range of motion (ROM) expressed as percentage relative to baseline; (**B**) Changes in limb circumference (CIR) at the mid-belly of the target muscle; (**C**) Changes in muscle stiffness assessed by acoustic radiation force impulse (ARFI) imaging.

Figure 3 displays the normalized changes in elbow flexor joint and muscle morphology from baseline (Pre) through D0 to D5 post-exercise, including (A) ROM, (B) CIR, and (C) muscle stiffness as assessed via ARFI. While no significant group × time interaction was detected (*p* > 0.05), within-group differences over time revealed distinct recovery profiles.

Both groups showed a reduction in ROM following MaxEc, with significant decreases observed from Pre to D0–D3 († or #, *p* < 0.05). However, the NL/NU group returned to baseline ROM by D4, whereas the C/NU group remained significantly below the baseline until D4. Circumference increased in both groups after exercise, peaking around D1–D2 and gradually returning toward baseline by D5. The C/NU group exhibited prolonged swelling (†), while the NL/NU group showed a more rapid resolution (#). Regarding muscle stiffness, both groups demonstrated elevated ARFI values post-exercise, with the NL/NU group peaking earlier (D2) and recovering to baseline (Pre) by D3, while the C/NU group remained elevated until D4. These trends suggest that the NL/NU group experienced faster morphological recovery, supporting the notion of a localized protective adaptation. This further reinforces the potential of IL-RBE across heterogeneous muscle groups.

### 3.3. Changes in Neuromuscular Control

**Figure 4:**
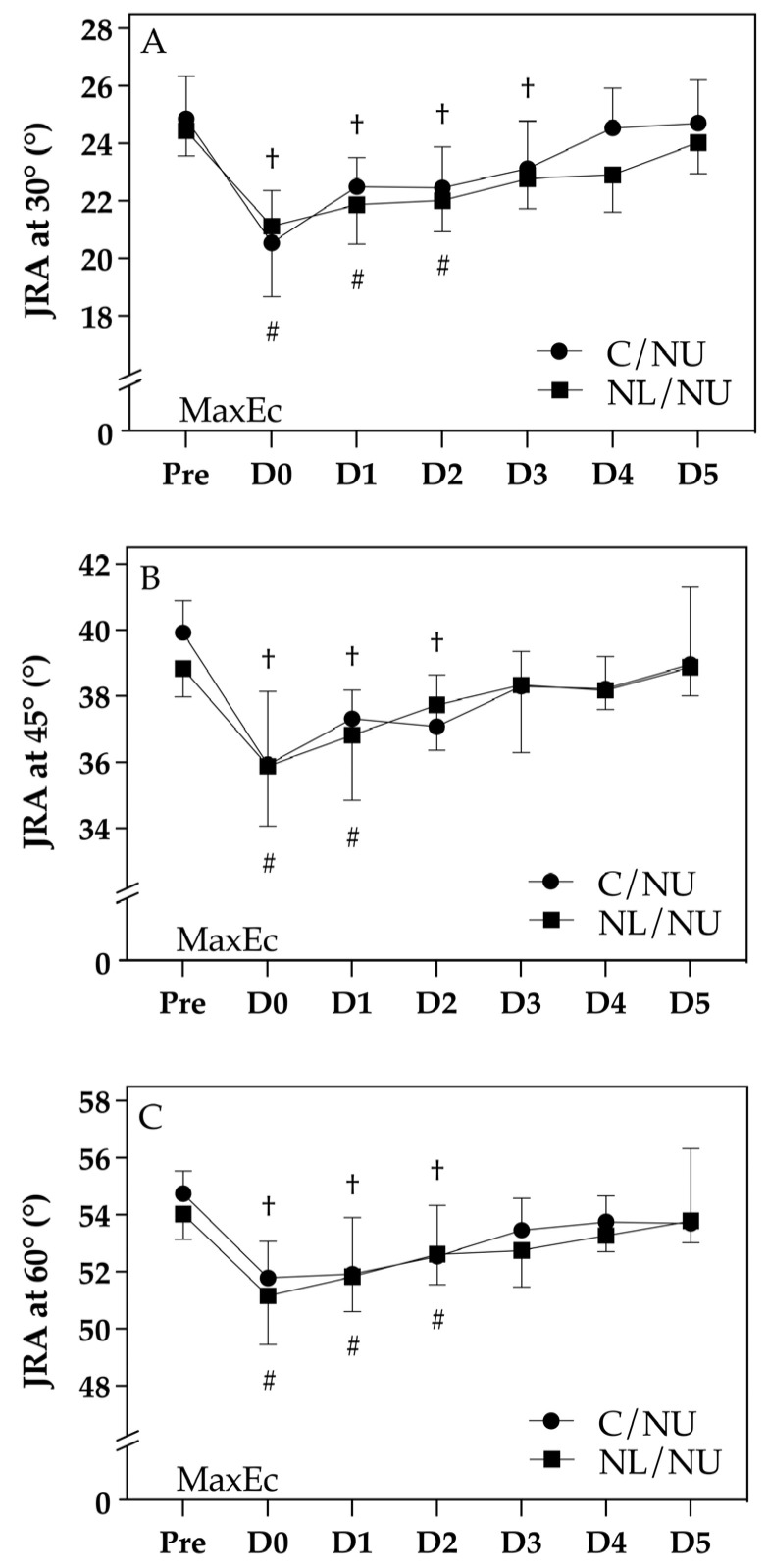
Neuromuscular control. Note: † indicates significant difference from Pre in the C/NU group (*p* < 0.05); # indicates significant difference from Pre in the NL/NU group (*p* < 0.05). C/NU = control followed by non-dominant upper limb eccentric exercise; NL/NU = non-dominant lower limb eccentric exercise followed by non-dominant upper limb eccentric exercise. MaxEc = maximal eccentric exercise. JRA = joint release angle. (**A**) JRA at 30°; (**B**) JRA at 45°; (**C**) JRA at 60°.

Figure 4 illustrates the temporal changes in neuromuscular control of the elbow flexors, as assessed by JRA at (A) 30°, (B) 45°, and (C) 60° across the Pre to D5 timeline. While no significant interaction effect between group and time was observed (*p* > 0.05), both groups exhibited significant within-group changes following MaxEc, with distinct recovery trajectories.

At 30°, both the NL/NU and C/NU groups demonstrated significant decreases in JRA at D0 and D1 († or #, *p* < 0.05), reflecting impaired proprioceptive control immediately post-exercise. However, the NL/NU group returned to baseline by D2, whereas the C/NU group remained significantly impaired until D3. Similar trends were seen at 45° and 60°, with both groups showing significant reductions in JRA post-exercise. Yet again, the NL/NU group showed a more rapid recovery, regaining pre-exercise JRA values earlier (by D3), while impairments persisted longer in the C/NU group (until D4 or D5).

These results suggest that the NL/NU group experienced faster restoration of neuromuscular function, supporting the existence of a localized IL-RBE. Prior eccentric loading of the lower limb may thus facilitate proprioceptive recovery in the ipsilateral upper limb following eccentric-induced muscle damage.

## 4. Discussion

This study investigated the presence of an ipsilateral repeated bout effect (IL-RBE) across heterogeneous muscle groups; specifically, whether eccentric exercise of the knee extensors would attenuate subsequent muscle damage in the ipsilateral elbow flexors. The NL/NU group, which received prior eccentric loading of the lower limb, demonstrated faster recovery of strength, joint morphology, and neuromuscular control in the elbow flexors compared to the control group (C/NU), despite similar baseline performance. These findings suggest that prior eccentric exercise in a remote but ipsilateral muscle group induces a protective adaptation, consistent with the concept of IL-RBE. The observed improvements in MVIC and concentric torque recovery align with classic RBE responses [3,15], and they extend the scope of such effects to an ipsilateral, non-homologous muscle group. Although previous studies primarily examined homologous or contralateral limb effects [8,10], our findings support the hypothesis that systemic and central mechanisms—rather than purely localized adaptations—contribute to repeated bout phenomena. Several previous studies have confirmed CL-RBE within homologous muscle groups such as bilateral elbow flexors or knee extensors [8,11]. However, few have investigated IL-RBE across functionally different muscle regions within the same limb. Tseng et al. (2020) and Green and Gabriel (2018) both demonstrated cross-education effects from unilateral eccentric training, but primarily in upper-to-upper or lower-to-lower limb paradigms [16,17]. In contrast, our study uniquely shows recovery enhancement from lower-limb eccentric preconditioning to ipsilateral upper-limb muscle damage, suggesting that the IL-RBE mechanism may generalize beyond anatomical or neural mirroring.

Among the potential mechanisms, neural adaptation and corticospinal excitability have been proposed as central contributors to the RBE [18]. Studies have demonstrated increased recruitment of higher-threshold motor units following eccentric training [19], as well as improved synchronization and firing efficiency that may reduce susceptibility to damage upon subsequent bouts. This neural priming may not be confined to the trained muscle alone, but may influence adjacent or ipsilateral musculature via interlimb transfer and central nervous system (CNS) plasticity [20]. In the current study, elbow flexor strength in the NL/NU group returned to baseline by day 3–4 post-exercise, whereas the C/NU group showed prolonged impairment until day 5. Similar trends were observed in range of motion (ROM), circumference, and muscle stiffness, indicating not only improved force output but also faster structural and inflammatory recovery. These findings are consistent with cross-education research demonstrating CNS-mediated, flow-on effects across limbs [21].

Interestingly, the earlier normalization of neuromuscular control (JRA) in the NL/NU group may reflect enhanced proprioceptive acuity and sensorimotor integration. Eccentric exercise often disrupts joint position sense (JPS) due to altered afferent signaling, but recent evidence suggests that unilateral eccentric training can improve JPS even in the untrained limb, likely through central nervous system adaptations [22,23]. Studies have shown enhanced sensorimotor cortex activity and cortical reorganization following unilateral eccentric exercise, supporting cross-limb proprioceptive benefits [16,17]. Unilateral eccentric training has been shown to improve proprioceptive acuity and neuromuscular control in the contralateral limb. This phenomenon is attributed to central nervous system adaptations, including increased cortical excitability and interhemispheric communication. For instance, Lee et al. (2009) demonstrated that unilateral strength training enhances the capacity of the motor cortex to drive homologous muscles in the untrained limb [24]. Additionally, Hadjisavvas et al. (2025) reported that eccentric exercise-induced fatigue affects proprioception and motor control in the upper limb, highlighting the systemic impact of such training [25]. These neural adaptations may involve increased excitability in motor areas, enhanced transcallosal signaling, and involvement of propriospinal pathways—all contributing to improved joint feedback and flow-on effects in motor control [26,27]. Collectively, these findings support the notion that faster JRA recovery in the NL/NU group is a result of centrally mediated proprioceptive enhancement following ipsilateral eccentric priming. One strength of this study is its focus on young women, a population underrepresented in previous IL-RBE literature. Although estradiol’s protective effects on muscle damage are minimal among women with regular menstrual cycles, hormonal and structural sex differences may still influence recovery kinetics, proprioception, and stiffness [28]. Our findings suggest that females also exhibit IL-RBE responses, supporting the applicability of eccentric preconditioning strategies across sexes. Future studies should include direct male–female comparisons and incorporate hormonal profiling to further clarify sex-specific mechanisms underlying RBEs.

Furthermore, the systemic inflammatory response may play a role. Post-exercise cytokines such as IL-6 and IL-10 have been shown to mediate anti-inflammatory adaptations in untrained limbs following eccentric exercise [29,30]. It is plausible that the initial knee extensor loading in our NL/NU group primed the systemic immune environment, reducing inflammatory burden and facilitating tissue repair in the subsequently trained elbow flexors. The observed effect may also reflect improved muscle–tendon unit compliance or remodeling of extracellular matrix (ECM) structures, as suggested by prior work on tendon stiffness and connective tissue remodeling post-eccentric exercise [31,32].

Not all prior research align with these findings. Some studies failed to observe protective effects when contralateral or heterologous muscles were involved [9,33], potentially due to differences in training volume, population, or eccentric intensity. Our study used a two-week inter-bout interval, which may be an optimal window for IL-RBE development, as RBEs have been shown to diminish if the interval exceeds 3–4 weeks [6]. Nevertheless, limitations should be acknowledged. The sample size was modest and restricted to young, healthy females. Further studies with larger, sex-diverse samples are needed to verify generalizability. Additionally, no mechanistic biomarkers (e.g., circulating cytokines, EMG frequency analysis) were directly measured, which limits the ability to confirm neuroimmune involvement. Additionally, although the study controlled for hormonal variation by including only eumenorrheic women and avoiding the early follicular phase, this may limit the generalizability of the findings to other phases of the menstrual cycle or individuals using hormonal contraception. Prior research has shown that menstrual cycle phase can influence neuromuscular performance and strength adaptation [14].

## 5. Conclusions

This study provides novel evidence of an ipsilateral heterologous repeated bout effect, demonstrating that prior eccentric loading of the knee extensors can attenuate muscle damage and accelerate recovery in the ipsilateral elbow flexors. Participants who underwent lower-limb eccentric preconditioning exhibited faster improvements in strength, joint mobility, muscle stiffness, and neuromuscular control compared to controls. These findings extend the application of RBE beyond traditional homologous or contralateral paradigms, highlighting the potential of remote, functionally unrelated muscle group conditioning within the same limb. Importantly, these results suggest that eccentric preconditioning of the lower limb may be applied in clinical rehabilitation protocols to promote recovery in ipsilateral upper-limb injuries—such as in cases of immobilization, disuse, or surgery—when direct training of the affected limb is not possible. Furthermore, this approach may benefit athletes by enhancing neuromuscular resilience and correcting unilateral overuse patterns. Future studies are warranted to validate these applications in clinical and athletic populations and to determine the optimal training dose and duration to elicit an IL-RBE. Such ipsilateral cross-adaptation may offer a promising strategy for optimizing rehabilitation and performance programming. Future research should investigate whether contralateral heterologous RBEs can be similarly induced, to further clarify the neural and systemic mechanisms governing cross-limb and cross-segment protective adaptations.

## Figures and Tables

**Table 1:** Descriptive statistics.

Independence Variable	Group	Pre	D0	D1	D2	D3	D4	D5
Strength Measurement								
MVIC Peak torque (Nm/kg)	C/NU	31.73 ± 5.08	17.09 ± 5.84 †	17.13 ± 3.43 †	19.95 ± 4.81 †	22.9 ± 3.67 †	24.49 ± 5.02 †	27.57 ± 3.46
	NL/NU	31.54 ± 7.04	16.26 ± 4.11 #	16.75 ± 5.11 #	19.57 ± 4.41 #	21.42 ± 4.47 #	25.30 ± 5.47 #	27.22 ± 5.53
ISOK Peak torque at 60°/s (Nm/kg)	C/NU	19.87 ± 3.85	11.12 ± 1.46 †	10.49 ± 2.01 †	12.85 ± 2.48 †	14.74 ± 3.15 †	16.14 ± 3.14 †	16.74 ± 3.15 †
	NL/NU	18.29 ± 4.88	11.05 ± 4.45 #	11.51 ± 4.62 #	12.55 ± 3.85 #	13.26 ± 4.82 #	15.38 ± 5.51 #	16.07 ± 6.46 #
ISOK Peak torque at 180°/s (Nm/kg)	C/NU	14.82 ± 1.39	10.24 ± 1.38 †	10.73 ± 1.45 †	11.03 ± 1.4 †	12.45 ± 2.19 †	13.97 ± 1.16	14.68 ± 1.49
	NL/NU	12.77 ± 2.13	10.09 ± 2.49 #	9.98 ± 2.25 #	10.42 ± 2.12 #	11.34 ± 2.62 #	12.18 ± 3.01	12.24 ± 2.83
Joint and Muscle Morphology Assessments								
Range of Motion (°)	C/NU	133.63 ± 8.23	122.63 ± 10.32 †	124.13 ± 11.73 †	122.63 ± 11.92 †	125.88 ± 9.96 †	128.50 ± 9.52 †	133.13 ± 8.69
	NL/NU	130.13 ± 5.33	115.25 ± 9.29 #	117.50 ± 8.60 #	120.00 ± 8.04 #	121.88 ± 7.90 #	125.00 ± 6.93 #	129.13 ± 5.38
Circumference (cm)	C/NU	25.11 ± 2.33	25.31 ± 2.28 †	25.36 ± 2.29 †	25.36 ± 2.32 †	25.31 ± 2.34 †	25.19 ± 2.28 †	25.15 ± 2.33
	NL/NU	26.19 ± 3.06	26.41 ± 3.03 #	26.46 ± 3.00 #	26.39 ± 2.98 #	26.29 ± 2.96 #	26.23 ± 3.01 #	26.18 ± 3.07
Muscle Stiffness (ARFI, m/s)	C/NU	0.95 ± 0.03	1.01 ± 0.05 †	1.06 ± 0.07 †	1.13 ± 0.10 †	1.15 ± 0.10 †	1.07 ± 0.06	0.96 ± 0.06
	NL/NU	0.96 ± 0.03	1.05 ± 0.06 #	1.13 ± 0.05 #	1.21 ± 0.05 #	1.14 ± 0.05	1.05 ± 0.06	1.02 ± 0.13
Neuromuscular Control								
JRA at 30° (°)	C/NU	24.88 ± 1.47	20.54 ± 1.83 †	22.5 ± 1.02 †	22.46 ± 1.42 †	23.13 ± 1.67 †	24.54 ± 1.38	24.71 ± 1.50
	NL/NU	24.46 ± 0.87	21.13 ± 2.43 #	21.88 ± 1.36 #	22.00 ± 1.10 #	22.79 ± 1.02	22.92 ± 1.31	24.04 ± 1.10
JRA at 45° (°)	C/NU	39.92 ± 0.97	35.92 ± 2.22 †	37.33 ± 0.87 †	37.08 ± 1.57 †	38.29 ± 1.08	38.21 ± 0.97	38.96 ± 2.34
	NL/NU	38.83 ± 0.87	35.88 ± 1.80 #	36.79 ± 1.97 #	37.75 ± 1.38	38.33 ± 2.05	38.17 ± 0.56	38.88 ± 0.87
JRA at 60° (°)	C/NU	54.75 ± 0.81	51.79 ± 1.28 †	51.92 ± 1.99 †	52.54 ± 1.79 †	53.46 ± 1.13	53.75 ± 0.90	53.71 ± 2.63
	NL/NU	54.04 ± 0.88	51.17 ± 1.69 #	51.83 ± 1.22 #	52.63 ± 1.06 #	52.75 ± 1.28	53.25 ± 0.56	53.79 ± 0.75

Note: † indicates significant difference from Pre in the C/NU group (*p* < 0.05); # indicates significant difference from Pre in the NL/NU group (*p* < 0.05). Abbreviations: MVIC = maximal voluntary isometric contraction; ISOK = maximal isokinetic concentric strength; ARFI = acoustic radiation force impulse imaging; JRA = joint release angle.

## Data Availability

The data presented in this study are available on request from the corresponding author. The data are not publicly available due to privacy.
